# Detection of human feces pecovirus in newly diagnosed HIV patients in Brazil

**DOI:** 10.1371/journal.pone.0272067

**Published:** 2022-09-06

**Authors:** Rodrigo Lopes Sanz Duro, Robson dos Santos Souza Marinho, Valquíria Lima Santana, Elis Muriel Marques Monti, Humberto Onias, Elaine Monteiro Matsuda, Ricardo Sobhie Diaz, Karina Rente Isidoro, Eric Delwart, Élcio Leal, Shirley Vasconcelos Komninakis

**Affiliations:** 1 Laboratório de Retrovirologia, Disciplina de Infectologia, Universidade Federal de São Paulo, São Paulo, Brazil; 2 Vigilância Epidemiológica, Prefeitura Municipal de Ferraz de Vasconcelos, São Paulo, Brazil; 3 Instituto de Ciências Biológicas, Brazil; 4 Programa de IST/AIDS de Santo André/SP, Santo André, Brazil; 5 Vitalant Research Institute, San Francisco, CA, United States of America; 6 Universidade Federal do Pará, Belém, Pará, Brazil; 7 Faculdade de Medicina (FMUSP)/Instituto de Medicina Tropical, Universidade de São Paulo, São Paulo, SP, Brazil; PLOS, UNITED KINGDOM

## Abstract

Circular single stranded DNA viruses (CRESS DNA) encoding a homologous replication-associated protein (REP) have been identified in most of eukaryotic groups. It is not clear yet the role in human diseases or details of the life cycle of these viruses. Recently, much interest has been raised in the evolutionary history of CRESS DNA owing to the increasing number of new sequences obtained by Next-Generation Sequencing (NGS) in distinct host species. In this study we describe two full-length CRESS DNA genomes obtained of two newly diagnosed HIV patients from São Paulo State, Brazil. The initial BLASTx search indicated that both sequences (named SP-FFB/2020 and SP-MJMS/2020) are highly similar (98%) to a previous CRESS DNA sequence detected in human fecal sample from Peru in 2016 and designated as pecovirus (Peruvian stool-associated circo-like virus). This study reported for the first time the Human feces pecovirus in the feces of two newly diagnosed HIV patients in Brazil. Our comparative analysis showed that although pecoviruses in South America share an identical genome structure they diverge and form distinct clades. Thus, we suggest the circulation of different species of pecoviruses in Latin America. Nevertheless, further studies must be done to examine the pathogenicity of this virus.

## Introduction

The single stranded DNA (ssDNA) viruses identified in eukaryotic hosts have a huge diversity, dispersed throughout ten families. Many of them are uncultured and remain unclassified [1]. Seven of these, namely, *Bacilladnaviridae*, *Circoviridae*, *Geminiviridae*, *Genomoviridae*, *Nanoviridae*, *Smacoviridae* and the proposed “*Redondoviridae*” family have small circular genomes, mostly encoding only two proteins: one for the genome replication (REP) initiation and the other for the capsid (CAP) [2]. Collectively, they were named as circular, Rep-encoding ssDNA (CRESS DNA) viruses [3–5]. The host range of these viruses includes plants (*Nanoviridae* and *Geminiviridae)*, fungi (*Genomoviridae*), algae (*Bacilladnaviridae*) and animals (*Circoviridae*, *Smacoviridae*, *Redondoviridae*) [6].

Phylogeny analysis of REP protein indicate two large clades representing classes of CRESS DNA viruses: (i) the first class (*Repensiviricetes*) includes the families *Geminiviridae* and *Genomoviridae*, and the unclassified clade CRESSV6 viruses; (ii) the second class (*Arfiviricetes*) includes the families *Bacilladnaviridae*, *Circoviridae*, *Nanoviridae* and *Smacoviridae*, and the unclassified clades CRESSV1 through CRESSV5 [4, 7].

The pecovirus (Peruvian stool-associated circo-like virus, PeCV) is an unclassified CRESS DNA virus. It has circular single stranded genome of approximately 2.9 kilobases, at least two putative proteins have been detected: REP and CAP. Because PeCV was recently identified by next-generation sequencing (NGS) in stool samples of children with gastroenteritis in Latin America, its role in human infection still needs to be addressed [8].

The extensive use of NGS on a variety of biological human samples showed that many viral agents are present in host samples other than the major known pathogenic viruses [3, 4, 7–9]. In addition, the host range of most CRESS DNA viruses detected by gut virome are debatable since most of them cannot be culture and the influence of viruses infecting the intestinal flora. Thus, the presence of these viruses in the human gut and the possible actions on it is not well understand.

Nevertheless, the assessment and characterization of these viral agents might be used as biological markers of microbiota equilibrium in health and disease [9].

The present study presents the identification and phylogenetic characterization of Human feces pecovirus sequences detected in newly diagnosed HIV patients. This is the first report of Human feces pecovirus in Brazil and this work will contribute with more information about this new viral agent in view of the great limitation given mainly with regard to the pathogenicity, epidemiology and molecular characteristics of the CRESS DNA viruses in human.

## Methods

### Study population and specimen collection

In this study, we analyzed a total of 37 fecal samples, 25 from newly diagnosed HIV and 12 HIV negative volunteers, attended to health units in the cities of Santo Andre, Ferraz de Vasconcelos and Sao Paulo, all in state of Sao Paulo, between 2019 and 2021. At the time of sample collection (within 10 days of starting treatment) the patients were under Combination Antiretroviral Therapy (cART) and showed no signs of diarrhea or gastroenteritis symptoms.

Inclusion criteria were all individuals aged 18 years or older, newly diagnosed HIV positive, sample collection would be performed if the patient was not receiving cART or had received it only for 10 days and the patients would not have other diseases. such as HCV and viral hepatitis. Not all newly diagnosed HIV patients agreed to participate in the study due to stool collection.

A control group with 12 fecal samples from HIV-negative voluntaries from city of Sao Paulo, State of Sao Paulo were included.

After collection, all the samples were stored at 4°C and within 24 hours were sent to Retrovirology Laboratory at UNIFESP, aliquoted in criotubes and stored at -80°C in the repository until the molecular analysis.

### Ethics statement

The study was conducted in accordance with the Declaration of Helsinki of 1975 (https://www.wma.net/what-we-do/medical-ethics/declaration-of-helsinki/), revised in 2013 and was approved by the Ethics Committees and the Institutional Review Board of the Federal University of São Paulo (UNIFESP) (CAAE#13652613.6.1001.5505). All participants signed the informed consent form after accepting to participate in this study.

### Viral metagenomics

The protocol used for NGS was according to Kapoor et al., 2008 with some modifications [10]. Briefly, 2g of feces were homogenized in 500μL of phosphate-buffered saline with 50uL of lysing matrix E (MP biomedicals). The centrifuged supernatant was filtered through the 0.45μm filter to remove bacteria and eukaryotic particles. The nucleic acids of the viral particles were enriched by filtration and treatment with a mixture of nucleases and the whole viral particles were purified with Magmax viral RNA isolation kit (Thermo Fisher Scientific). Reverse transcription reaction with SuperSript III Reverse Transcriptase (Thermo Fisher Scientific) was used and a complementary strand was synthesized with Klenow fragment polymerase (Uniscience do Brasil). The library, with 37 samples, was prepared using the Nextera XT DNA Sample Preparation Kit (Illumina, Inc) according to the manufacturer´s instructions. It was sequenced using Miseq sequencer (Illumina, Inc) to generate 2X 300bp pair-end reads. After trimming, the sequences were sent to Dr. Eric Delwart Laboratory at the University of California (UCSF/Vitalant Institute) to be analyzed according to the pipeline developed by Deng Xutao *et al*., 2014 [11]. To analyze a possible similarity with viral sequences, singlets and contigs generated were translated using BLASTx. Only sequences with E score <10^−5^ were selected. The sequences found were used as a reference for the mapping carried out on Geneious R9 (Biomatters Ltd).

To confirm the data all the procedure was repeated from the beginning in a different laboratory, in another institution. Different aliquots of feces were used for nucleic acid extraction and a new library was prepared, and sequenced in a different Miseq sequencer equipment. No significant differences between the two runs were observed, only differences in the number of the reads (data not shown). In all experiments water was used as a negative control, including the NGS.

### Alignments and annotation

The resulting contigs were subjected to a modified protein BLAST search using Ugene software [12] to identify novel members of the CRESS DNA viruses. Based on the best results (best hits) from BLASTx search, genomes of PeCV and other related viruses were chosen for further analysis. Next full or nearly full genomes were aligned using MAFFT software [13]. Genome annotation was performed using Gatu software [14] and the PeCV sequence KT600065 as reference. The translated sequences were used to determine viral motifs in the online server Motif Finder (https://www.genome.jp/tools/motif/).

### Genetic identity

Genetic distance and their standard error were calculated using maximum composite likelihood model plus gamma correction and bootstrap with 100 replicates. Distances were calculated using MEGA software (Version X) [15]. To estimate the similarity of sequences we used a pair-wise method implemented in the program SDT [16]. To estimate the similarity alignments of every unique pair of sequences were done using algorithms implemented in MUSCLE [17]. After the computation of the identity score for each pair of sequences the program then uses the NEIGHBOR component of PHYLIP to compute a tree [18]. The rooted neighbor-joining phylogenetic tree orders all sequences according to their likely degrees of evolutionary relatedness. Results are presented in a frequency distribution of pairwise-identities in a graphical interface.

### Phylogenetic analysis

Phylogenetic trees of CRESS DNA virus genomes were constructed using the maximum likelihood approach. To obtain reproducible results and provide greater reliability of clustering pattern of trees the statistical support of branches was evaluated by approximate likelihood ratio test (aLRT). Trees were inferred using the FastTree [19] software and the GTR model plus gamma distribution and the proportion of invariable sites were used. Selection of best model was done according to the likelihood ratio test (LRT) implemented in the jModeltest software [20].

## Results

### Metagenomics overview and clinical features of HIV patients

A total of 11,830,712 sequence reads were yield with an average of 622,699 reads per sample. The unclassified PeCV was identified (272 hits) in two samples from patients SP-FFB/2020 and SP-MJMS/2020. The Genbank accession number of the sequences are MZ394713 and MZ394714. In the 12 HIV-negative samples, viruses other than PeCV were found (data not shown). Patient samples showed few sequence reads with low BLASTx hits for the pathogenic viral families commonly found in gut. In the SP-MJMS/2020 sample, there were hits for Human Immunodeficiency Virus (HIV) with a high percentage of similarity to the HIV-1 gp120. The hits for Simian-Human Immunodeficiency virus, Murine Leukemia related retroviruses and Murine leukemia virus may have been generated due to high similarity between the genomes of the members of the *Retroviridae* family. The SP-FFB / 2020 sample had no hits for the *Retroviridae* family. There were for SP-FFB/2020 19, 765 (3%) sequence reads for bacteria, 6,588 (1%) for humans, for SP-MJMS/2020 33,572 (6%) for bacteria and 16,786 (3%) for human reads. Patients SP-FFB/2020 and SP-MJMS/2020 had CD4^+^ T cell count of 691 cells/mm^3^ and 752 cells/mm^3^ with HIV viral loads of 290, 976 copies/mL (5.46 log) and below the limit of detection (<50 copies/mL); both with diagnoses of syphilis without any AIDS-defining disease.

### Genome organization of Brazilian pecoviruses

**Fig 1:**
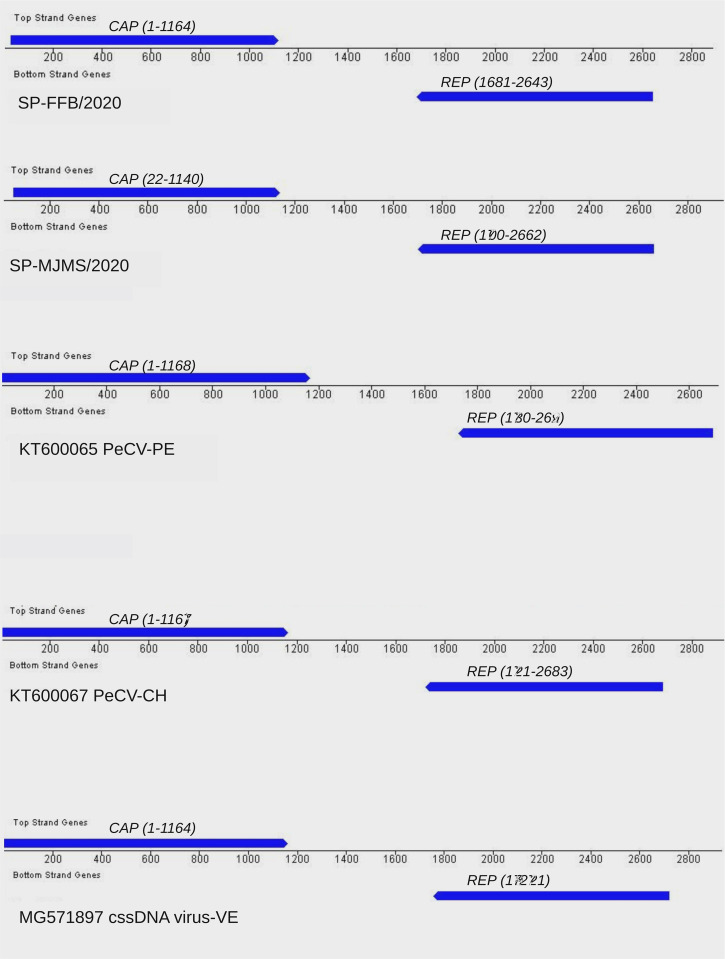
Annotated genomes of PeCV. Each diagram represents the annotation of one sequence. Lines represent genome position and blue are indicating ORFs CAP and REP in opposite orientation. Numbers within parenthesis indicate the location of ORFs in each sequence. Diagrams were constructed with Gatu software.

Based on the best hits of BLASTx search (megablast) we compared our sequences with PeCV detected from previous studies (i.e., KT600065, KT600066 and KT600067) and others CRESS DNA human viruses (MG571895 and MG5718957). The sequence with highest similarity was KT600065 which was detected in Peru by metagenomic analysis in 2013. This sequence was used as a reference to perform the annotation of the Brazilian sequences. Our sequences, denoted SP-FFB/2020 and SP-MJMS/2020, contained 2887 and 2943 bases with a CG content of 49.79% and 49.49% respectively. We found two potential ORFs in opposite orientation and the location of putative proteins CAP and REP is indicated in the Fig 1. Brazilian sequences also have the canonical PeCV nonamer TTTTATGAG which form a stem-loop (not shown). For illustrative purposes the genome maps of other PeCV are also shown in the Fig 1. Although the genome organization of PeCV are quite similar their genetic divergences are very high (see the estimation of similarities and genetic distances below).

**Fig 2:**
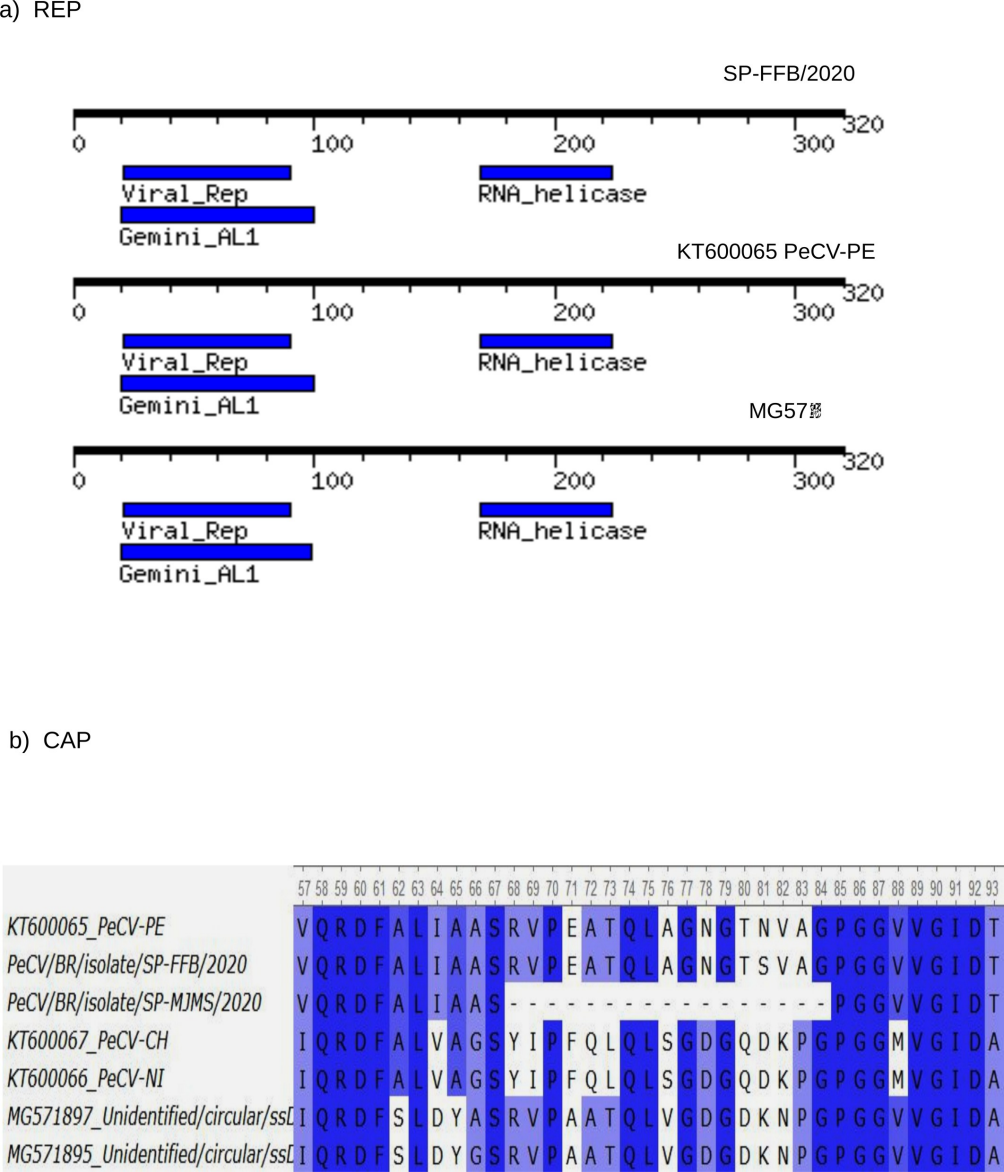
Characteristics of REP and CAP of PeCV. (**a**) The diagram represents the REP region of PeCV (black line) and the main motifs detected by motif finder analysis (blue areas) using pfam data base. Motifs were determined online by the server MotifFinder (https://www.genome.jp/tools/motif/) b) Amino acid alignment of the CAP region of PeCV. Residues in dark blue are those conserved, sites with some variations are indicated by light blue and white. Dashes indicate gaps in the SP-MJMS/2020 sequences.

The translated ORF REP revealed the presence of two rolling-circle replication motifs II (^46^VHWH^49^) and III (^87^YVKK^90^) [3]. The helicase motifs Walker A (^172^GRAGSGKS^179^) and Walker B (^214^IWFDEF^219^) were also found in the Brazilian PeCV genomes [3, 4]. To better explore the details of PeCV genomes we search pfma protein database and compared motifs detected in the ORF REP (Fig 2A). We found in the Brazilian sequences the following motifs: I) PF00799, Geminivirus REP catalytic domain (pfam ID: PF00799) location 20–100 and II) RNA helicase (pfam ID: PF00910), genome location 169–224. For illustrative purpose the motifs of other PeCV are also indicate in the figure. The translated ORF CAP indicate little variations of residues the exception was the region 68 through 84 which is missing in the Brazilian sequence SP-MJMS/2020. The complete alignment of CAP protein is shown in Fig 2B.

### Phylogenetic analysis of complete genomes of pecovirus

**Fig 3:**
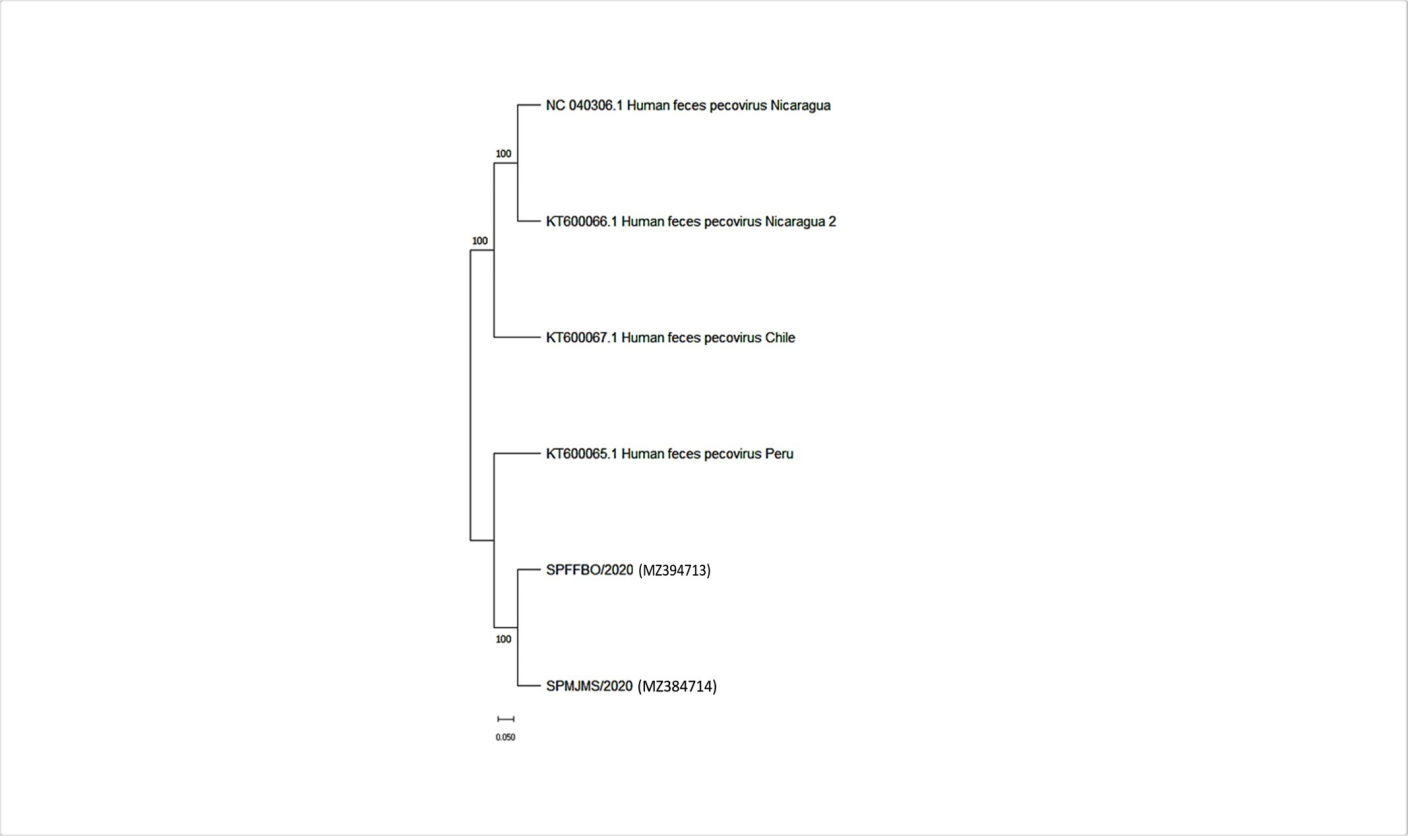
The construction of the phylogenetic tree was performed using the Maximum Likelihood test model. In addition, we used the GTR+ gamma correction and the proportion of the invariant sites model, as selected by the Modeltest software. Branch support was achieved by approximate likelihood ratio test (aLRT) and is shown in a color scale. Pecoviruses identified in this study are highlighted in gray. Hosts in which viruses were identified are indicated in colors.

A detailed phylogenetic analysis was performed to determine the relatedness of Brazilian sequences with other PeCV, smacoviruses, cycloviruses and circoviruses viruses detected in distinct hosts. The maximum likelihood tree constructed with viral genomes show that Brazilian sequences generated in this study are monophyletic and are related to the sequence KT600065, detected in Peru in 2013 showed in Fig 3. The Sequences of PeCV are distributed in two divergent clades: one composed by the Brazilian sequences and Peru sequences (KT600065) and another composed by sequences identified previously in Nicaragua (KT600066) and Chile (KT600067). The genetic distance of these clades is 5%.

### Variability of CAP and REP in members of CRESS DNA viruses

**Fig 4:**
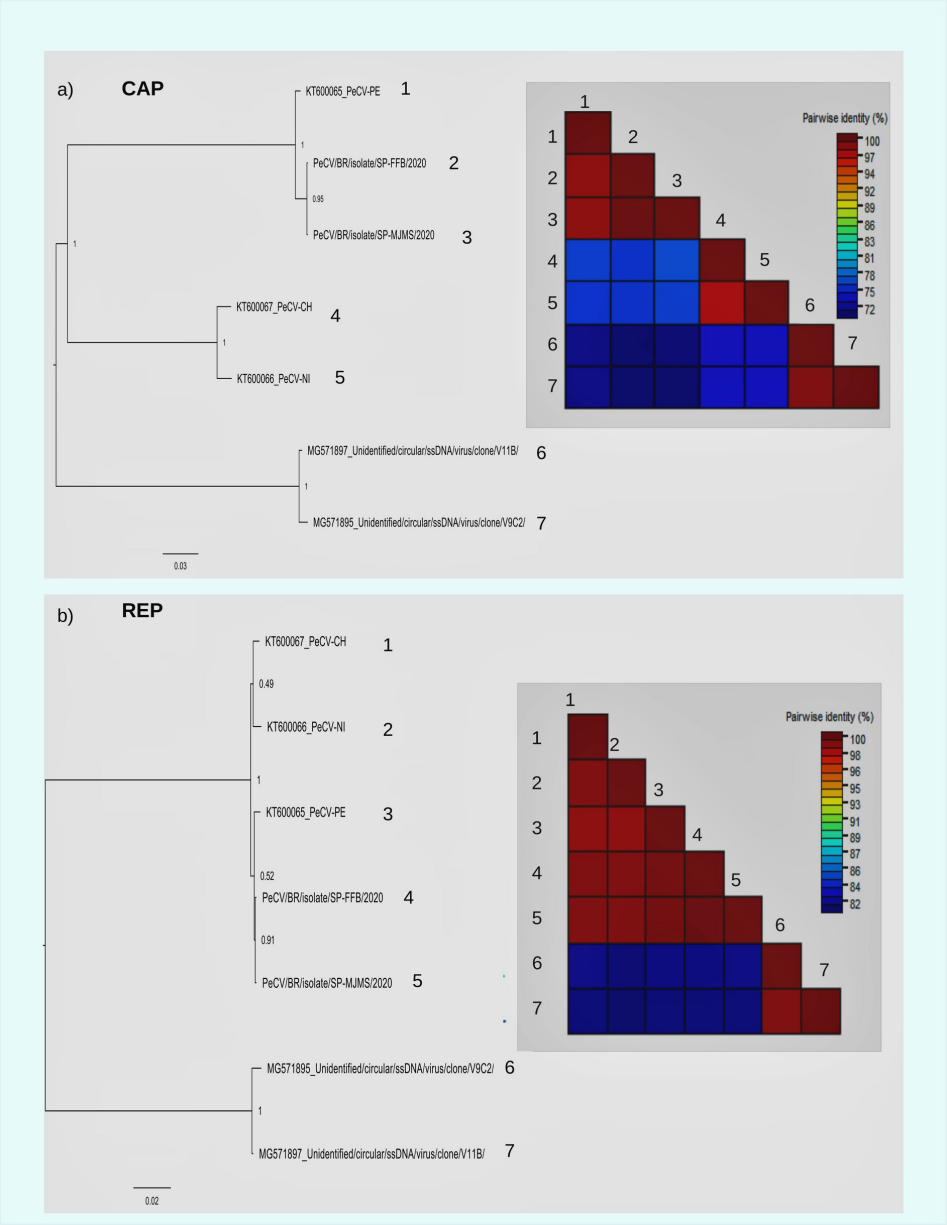
Similarity of CAP and REP proteins in members of CRESS DNA viruses. Diagrams indicate the similarity of the translated CAP (a) and REP (b) proteins in members of pecoviruses and circoviruses. The similarity percentages are indicated by colors according to the scale pattern indicated in each diagram. Maximum likelihood tree of genome of pecovirus and circoviruses. Tree was inferred in FastTree using GTR+ gamma correction and the proportion of invariable sites model as selected by the jModeltest software. Branch support was achieved by approximate likelihood ratio test (aLRT) and is shown in a color scale.

We performed a similarity analysis comparing Brazilian sequences with some members of pecoviruses, smacovirus, circovirus and cycloviruses previously described [1]. In the CAP region we found a similarity between Brazilian sequences and the reference sequence that (KT600065) reaches 98%, but the similarity between sequences from Brazil, Peru, Chile and Nicaragua were only 71%. This may indicate the presence of distinct genotypes in Latin American countries. The pairwise similarity of all sequences is indicated in the diagrams of Fig 4.

## Discussion

We report the first identification and characterization of Human feces pecovirus in fecal samples from two newly diagnosed HIV patients in Brazil. The identified genomes had 2887 and 2943 bases with a similar genomic structure to the CRESS DNA genomes, with ORFs for the REP and CAP proteins, the canonical PeCV nonamer, rolling circular replication motifs and helicase motifs.

There was a high similarity (98%) with the sequence of the PeCV KT600065 (98% similarity) previously identified in Peru in samples of children with diarrhea. On the other hand, our sequences showed 71% similarity with sequences from Chile and Nicaragua [8]. Thus, we can suggest the presence of distinct genotypes or species that would be circulating in Latin America. The circulation of different species has also been suggested by other authors who recently identified PeCV in Cameroon in a sample of individuals with symptoms of gastroenteritis [21] and that the REP protein had 31% to 42% aa sequence identity for all other genes Rep.

In addition, when based on CAP protein this similarity was 22% to 42% to all other PeCV and clustered distantly from pigs and human strains. The authors suggested that there are multiple species of this clade and a high genetic diversity in the Cameroonian population [21]. REP protein is the most conserved at amino acid level among CRESS DNA and can differentiate new viruses from those described [22].

We observed in our study a deletion of 19 amino acids in the CAP protein, in the sample SP-MJMS/2020, which could indicate that this region is more prone to deletions.

The members of different taxa of CRESS DNA viruses have a rapid evolution due to the high rates of substitution/site/year and their recombination mechanisms can be compared to RNA viruses [23]. However, notwithstanding this rapid evolution, there is a conserved predicted disorder, that does not compromise its function and which explains the success of CRESS DNA in different environments [22].

Despite the complexity of CRESS DNA viruses, through high throughput sequencing, it has been possible to demonstrate the ability of their spread in different environments and hosts (3). The viral metagenomics has shown in recent years the description of eukaryotic ssDNA in healthy individuals, which may be part of the virome present in the gut in humans [24]. The PeCV genomes of this study were detected in two newly diagnosed HIV positive patients. These patients had no AIDS-defining illness, without diarrhea or any symptoms of gastroenteritis.

In previous studies PeCV was identified in adult or children during events of diarrhea or outbreaks of acute gastroenteritis [8, 21–25]. It is known that people living with HIV/AIDS (PLWHA) show impaired immune system and that even after treatment there is a residual viral replication in some tissues, a chronic inflammation and persistent immune activation [26]. One of the primary sites of HIV replication in the initial stages is the gut-associated lymphoid tissue (GALT). This tissue is important in the HIV pathogenesis, playing a role in the immunological activation and HIV progression (Thompson *et al*, 2017). It is one of the main sites for HIV seeding and the establishment of infection [26]. As a main immunological regulator, GALT alterations could influence the gut environment and the dynamics of bacterial and viral populations in the gut [27], which could explain the occurrence of the pathogenic viruses found in the two patients.

In fecal samples analyzed from Ugandan HIV patients in different clinical status and HIV negative individuals, the authors detected viruses from the families *Adenoviridae*, *Anelloviridae*, *Circoviridae* and *Papillomaviridae* and observed an expansion of Adenoviruses and Anelloviruses in the patients of the group with severe immunodeficiency [28]. In fact, the viral families described in our study have been described in previous studies and, in most cases, causing gastroenteritis and diarrhea in children or HIV-positive patients with immunodeficiency state [29–32].

It is known that PeCV are capable of recognition by the human immune system, and as so, the immune system could interfere in the PeCV replication [33]. The patients were PeCV was detected are in the beginning of cART but both had normal CD4^+^ T cell counts. This is an indication that immune system of these patients are relatively intact and could clear viral infections, which could be answer for the absence of symptons.

The detection of HIV in feces has been previously described in samples from chronically infected HIV patients with detectable viral load. The GALT is one of the largest HIV reservoirs. Infected CD4^+^ T cells move from GALT to intestinal lumen through diapedesis increasing the viral detection in feces [33]. This could explain the fact that we identified HIV in both patients despite detectable and undetectable viral loads.

The study had some limitations, such as not using stool samples from a control group, which could be individuals without HIV infection and with diarrhea and gastroenteritis; the samples were not tested by polymerase chain reaction (PCR) with PeCV specific primers. In addition, we have not tested a large number of samples from individuals in different clinical status that could show whether PeCV can lead to diseases in the gut, potentiate the disease caused by other pathogenic intestinal viruses or if it is part of a normal intestinal microbiota. This study reported for the first time the PeCV, a member of CRESS DNA viruses, in the feces of two newly diagnosed HIV patients in Brazil and we suggest the circulation of different species of this virus in South America.
